# A Novel Dynamic Three-Level Tracking Controller for Mobile Robots Considering Actuators and Power Stage Subsystems: Experimental Assessment

**DOI:** 10.3390/s20174959

**Published:** 2020-09-02

**Authors:** José Rafael García-Sánchez, Salvador Tavera-Mosqueda, Ramón Silva-Ortigoza, Victor Manuel Hernández-Guzmán, Magdalena Marciano-Melchor, José de Jesús Rubio, Mario Ponce-Silva, Miguel Hernández-Bolaños, Jesús Martínez-Martínez

**Affiliations:** 1 División de Ingeniería Mecatrónica, Tecnológico de Estudios Superiores de Huixquilucan, Tecnológico Nacional de México, Estado de México 52773, Mexico; jose.g.s@huixquilucan.tecnm.mx (J.R.G.-S.); division.mecatronica@huixquilucan.tecnm.mx (J.M.-M.); 2 Laboratorio de Mecatrónica & Energía Renovable, Centro de Innovación y Desarrollo Tecnológico en Cómputo, Instituto Politécnico Nacional, Ciudad de México 07700, Mexico; staveram1500@alumno.ipn.mx (S.T.-M.); mmarciano@ipn.mx (M.M.-M.); mbolanos@ipn.mx (M.H.-B.); 3 Facultad de Ingeniería, Universidad Autónoma de Querétaro, Querétaro 76010, Mexico; vmhg@uaq.mx; 4 Sección de Estudios de Posgrado e Investigación, ESIME Azcapotzalco, Instituto Politécnico Nacional, Ciudad de México 02250, Mexico; jrubioa@ipn.mx; 5 Departamento de Ingeniería Electrónica, CENIDET, Tecnológico Nacional de México, Morelos 62490, Mexico; mario.ps@cenidet.tecnm.mx

**Keywords:** mobile robots, DC motors, DC/DC power converters, control design, trajectory tracking, dynamic three-level controller, hierarchical controller, PI current control, differential flatness

## Abstract

In order to solve the trajectory tracking task in a wheeled mobile robot (WMR), a dynamic three-level controller is presented in this paper. The controller considers the mechanical structure, actuators, and power stage subsystems. Such a controller is designed as follows: At the high level is a dynamic control for the WMR (differential drive type). At the medium level is a PI current control for the actuators (DC motors). Lastly, at the low level is a differential flatness-based control for the power stage (DC/DC Buck power converters). The feasibility, robustness, and performance in closed-loop of the proposed controller are validated on a DDWMR prototype through Matlab-Simulink, the real-time interface ControlDesk, and a DS1104 board. The obtained results are experimentally assessed with a hierarchical tracking controller, recently reported in literature, that was also designed on the basis of the mechanical structure, actuators, and power stage subsystems. Although both controllers are robust when parametric disturbances are taken into account, the dynamic three-level tracking controller presented in this paper is better than the hierarchical tracking controller reported in literature.

## 1. Introduction

Differential drive wheeled mobile robots (DDWMRs) have been intensively studied by the control community over the last few decades [1,2]. Since these kind of systems are underactuated and they are restricted in their lateral motion [3], any control task becomes a real challenge. In this sense, four control tasks have been detected on the specialized literature [4,5,6]: regulation, path following, obstacle avoidance, and trajectory tracking, the latter being the most studied due to its practical applications. In this context, the dynamical model of a DDWMR is composed by three subsystems, i.e., mechanical structure, actuators, and power stage. In this regard, the great majority of the published papers only consider the mathematical model, kinematic or dynamic, associated with the mechanical structure and, at most, the dynamics of the actuators. However, with the aim of achieving a better performance in the DDWMR, recent papers have also included the dynamics of the power stage [7,8,9,10]. It is worth mentioning that considering the dynamics of the electric power supply renders the control algorithm complex; but perhaps the performance of the DDWMR might be improved (for mechatronic systems [11,12,13,14]).

Based on the aforementioned, when designing tracking controls for DDWMRs two directions have been taken, both related to the mathematical model of the mechanical structure. These are, when the control design is performed on the basis of: (i) the kinematic model or (ii) the dynamic model. Thus, the following review highlights the papers that, additionally, take into account the dynamical models of the actuators and the power stage subsystems. It is important to emphasize that this review only contemplates papers published in the last 12 months. Complementary works can be found in the literature review reported in [9,10].

### 1.1. Control Strategies Based on the Kinematic Model

Papers considering the kinematics of the mechanical structure in control design can be divided in turn into three paths: (1) kinematics of the mechanical structure, (2) kinematics of the mechanical structure plus dynamics of the actuators, and (3) kinematics of the mechanical structure plus dynamics of the actuators and power stage. These approaches are described below.

#### 1.1.1. Kinematics of the Mechanical Structure

The most common way of solving the tracking task in a DDWMR is by using only the kinematics of the mechanical structure. For example, an adaptive visual control was presented by Li et al. in [15]. Xie et al. in [16] proposed a kinematic control combined with a dynamic compensator that achieves asymptotic stability. A sliding mode control (SMC) with double control loop was reported by Seo et al. in [17]. Boubezoula et al. in [18] designed a SMC for the flat outputs of the DDWMR and by proposing an adaptive gain discontinuous control law a zero error convergence is achieved. On the other hand, Miao et al. in [19] introduced a leader-follower control strategy based on a distributed estimation law for each follower, with the aim of estimating the vector state of the leader, and a distributed formation control law that uses the estimated information of the followers and the formation error. In [20], Zhang et al. developed a discrete-time domain control that considers the lost of data over an ideal communication network and two algorithms for compensate the delays over that same network. In [21], Zhang et al. presented a vision-based control and an adaptive continuous controller for solving the tracking and regulation tasks on the DDWMR. Roy et al. in [22] proposed an adaptive switching gain-based robust control that considers linear parametric uncertainty, whose efficacy was experimentally assessed with an adaptive SMC. Cui et al. in [23] reported a robust control algorithm for solving the tracking and obstacle avoidance tasks that uses an adaptive unscented Kalman filter for estimating the slippage of the wheels and an unscented Kalman filter for adjusting the covariance of the noise generated by the slippage estimation process. In [24], Dönmez et al. designed a visual servoing go-to-goal behavior controller to steer the DDWMR to a static target; such a controller was also experimentally assessed with two controls, a PID control and a fuzzy-PID control. Melo et al. in [25] introduced a leader-follower SMC that considers parametric uncertainty and uses a fuzzy adaptive formation technique so that the parameters associated with perturbations are not required. Falsafi et al. in [26] developed an optimal fuzzy logic controller that considers velocity and acceleration restrictions imposed by the mathematical model of the DDWMR. Additionally, the performance of the controller was assessed with a fuzzy controller and with a model predictive controller via simulations performed in Matlab. In [27], Bai et al. presented a model predictive control based on tire mechanics that prevents the sideslip and improves the performance of the DDWMR when solving the tracking task. The proposed control was assessed with a kinematic model predictive control. Wang et al. in [28] proposed a smooth controller with time-varying feedback parameters that solves the tracking and the stabilization tasks. Lastly, Guechi et al. in [29] solved the trajectory tracking problem by designing a novel fuzzy predictor observer for dealing with the delay measurements and a parallel-distributed compensation control for the mechanical structure.

#### 1.1.2. Kinematics of the Mechanical Structure Plus Dynamics of the Actuators

When the kinematics of the mechanical structure and the dynamics of the actuators are considered in control design, the performance of a wheeled mobile robot (WMR) is significantly improved [30,31,32]. In this direction, Márquez-Sánchez et al. in [33] reported and implemented on an embedded hardware a two-level tracking control composed by a kinematic control for the mechanical structure and a PI control for the actuators.

#### 1.1.3. Kinematics of the Mechanical Structure Plus Dynamics of the Actuators and Power Stage

According with literature, there are only four papers that have considered together the kinematics of the mechanical structure, the dynamics of the actuators and the power stage in control design. All these approaches were based on the hierarchical control concept, where three levels of control, one for each subsystem, were proposed. For example, Silva-Ortigoza et al. in [7] reported the first three-level switched tracking controller that considered the kinematics of the mechanical structure and the dynamics of the actuators and the power stage. García-Sánchez et al. in [8] designed a three-stage average tracking controller that also used the models of the same three subsystems. In such a paper, an assessment of the controller was done when its switched implementation is carried out via PWM or Σ−Δ-modulation. Later, García-Sánchez et al. in [9] introduced a new three-level average tracking controller that also took into account the three subsystems. In that paper, the developed controller was experimentally assessed with a controller that neglects the dynamics of the power stage. Lastly, García-Sánchez et al. in [10] presented a new three-level switched tracking controller whose performance was experimentally assessed with the three-level average controller reported in [9].

### 1.2. Control Strategies Based on the Dynamic Model

Papers considering the dynamics of the mechanical structure subsystem in control design, can be divided in two paths: (1) dynamics of the mechanical structure and (2) dynamics of the mechanical structure plus dynamics of the actuators. These approaches are presented below.

#### 1.2.1. Dynamics of the Mechanical Structure

By using the dynamics of the mechanical structure for control design purposes, robust algorithms can be easily designed [34]. Also, as stated in [35], a more realistic scenario is achieved when the torque is used as the control input. In this direction, tracking controls that consider only the dynamics of the mechanical structure are presented here. Nguyen et al. in [36] proposed a neural network-based adaptive SMC that contemplates the slipping between the wheels and the ground and uncertainties in the mathematical model. In [37], Mirzaeinejad and Shafei reported a predictive control whose performance was assessed with a SMC. Huang et al. designed in [38] a neural-network tracking control that was robust to parametric uncertainties.

#### 1.2.2. Dynamics of the Mechanical Structure Plus Dynamics of the Actuators

Controls that take into account the dynamics of the mechanical structure plus the actuators in its design can solve the tracking task in a more efficient way. However, the algorithm turns out to be more complex. In this context, Kumar et al. in [39] introduced a hierarchical controller based on SMC and PI control for the kinematic model and a PID control for the dynamics and the actuators. In [40], Mirzaeinejad developed a robust optimization-based nonlinear control, whose feasibility was assessed with a SMC.

### 1.3. Discussion of the Related Literature, Motivation, and Contribution

The literature provided herein shows that five approaches have been naturally generated when proposing tracking control algorithms for DDWMRs. These approaches are linked to the mathematical model of the mechanical structure, as briefly shows the Table 1. In such a table, the acronyms MS, A, and PS stand for Mechanical Structure, Actuators, and Power Stage, respectively.

As it can be observed in Table 1, when the dynamics of the mechanical structure subsystem is used for control design purposes, at most the dynamics of the actuators subsystem has been also introduced. However, the dynamics of the power stage subsystem has been neglected.

Motivated by the literature previously presented, see Table 1, the contribution of this paper is twofold:

- To design a dynamic tracking controller that uses the dynamics of the mechanical structure, actuators, and power stage subsystems. The proposed controller has three levels: at the high level, for the mechanical structure, is a dynamic control; at the medium level, for the actuators, are two PI current controls; at the low level, for the power stage, are two controls based on differential flatness.
- To experimentally validate the proposed controller and, in order to highlight the contribution of this paper, to assess its performance with results of the hierarchical tracking controller reported in [9]. This latter was designed on the basis of considering the kinematic model of the mechanical structure and the dynamics of the actuators and power stage.

The rest of this paper is structured as follows. The dynamic tracking controller is designed in Section 2. The experimental results of the proposed approach and the assessment with results of a controller previously reported in literature are presented in Section 3. Finally, some conclusions are given in Section 4.

## 2. Dynamic Three-Level Tracking Controller that Considers Actuators and Power Stage Subsystems

### 2.1. Generalities of the DDWMR

**Figure 1:**
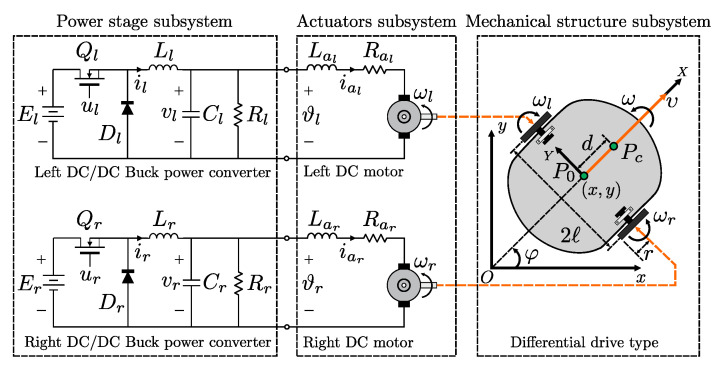
Subsystems composing the DDWMR under study.

The DDWMR is presented in Figure 1 and is composed by three subsystems: mechanical structure, actuators, and power stage. In such a figure, and in the rest of this paper, the components of the actuators and the power stage linked to the right and the left wheels are specified through the subscripts *r* and *l*, respectively. In the following, each subsystem is described.

- Mechanical structure. It is a differential drive type where υ and ω are the straight linear and angular velocities, φ is the heading angle of the DDWMR, $\omega_{r}$ and $\omega_{l}$ correspond to the angular velocities of the right and left driving wheels, respectively. The width of the DDWMR is 2ℓ and *r* is the radius of each wheel. The origin of the xy world coordinate system is *O* and $P_{0}$ is the origin of the XY coordinate system fixed to the DDWMR. Additionally, $P_{0}$ is placed at the middle of the axis that is common to the left and right driving wheels. The center of mass of the DDWMR is $P_{c}$, which is on the X− axis, and the distance from $P_{0}$ to $P_{c}$ is *d*. For the latter description, $m_{c}$ and $m_{w}$ are the mass of the body and wheel with a motor, respectively. $I_{c}$, $I_{w}$, and $I_{m}$ are the moment of inertia of the body about the vertical axis through $P_{c}$, the wheel with a motor about the wheel axis, and the wheel with a motor about the wheel diameter, respectively.
- Actuators. Two DC motors are employed, one for each wheel, whose parameters $\left(L_{a_{r}},L_{a_{l}}\right)$ and $\left(R_{a_{r}},R_{a_{l}}\right)$ correspond to the inductances and the resistances of armature, whereas $\left(i_{a_{r}},i_{a_{l}}\right)$, $\left(\vartheta_{r},\vartheta_{l}\right)$ and $\left(\omega_{r},\omega_{l}\right)$ are the currents, the applied voltages at the armature terminals, and the angular velocities of the shafts. Other parameters are $\left(J_{r},J_{l}\right)$, $\left(k_{e_{r}},k_{e_{l}}\right)$, $\left(k_{m_{r}},k_{m_{l}}\right)$, and $\left(b_{r},b_{l}\right)$, associated with the moments of inertia related to the rotors and motor loads, the counterelectromotive force constants, the motor torque constants, and the viscous friction coefficients, respectively.
- Power stage. This subsystem is made up of two DC/DC Buck power converters, one for each motor. Here, $\left(E_{r},E_{l}\right)$ are the power supplies, $\left(u_{r},u_{l}\right)$ are the switched control signals that regulate the voltages $\left(v_{r},v_{l}\right)$ at the terminals of the capacitors $\left(C_{r},C_{l}\right)$ and the loads $\left(R_{r},R_{l}\right)$ through the transistors $\left(Q_{r},Q_{l}\right)$ and the diodes $\left(D_{r},D_{l}\right)$, while $\left(i_{r},i_{l}\right)$ are the currents that flow in the inductances $\left(L_{r},L_{l}\right)$.

### 2.2. Design of the Dynamic Three-Level Tracking Controller

This section presents the design of a dynamic three-level tracking controller for a DDWMR. The proposed controller introduces in its design the dynamics of the three subsystems mechanical structure, actuators, and power stage. The structure of this controller is described below.

1. High-level control. It comprises a kinematic control $\left(\upsilon_{f},\omega_{f}\right)$ ensuring $\left(x,y,\varphi \right)\to \left(x^{*},y^{*},\varphi^{*}\right)$ and a computed-torque control $\left(\tau_{r},\tau_{l}\right)$ ensuring $\left(\omega_{r},\omega_{l}\right)\to \left(\omega_{r}^{*},\omega_{l}^{*}\right)$, i.e., that $\left(\upsilon ,\omega \right)\to \left(\upsilon_{f},\omega_{f}\right)$. The latter allows to compute the desired armature current to be tracked by each actuator of the DDWMR.
2. Medium-level control. Here, two PI current controls are proposed. These controllers ensure that electric current through the motor armature circuits reach their required values to generate the desired torque that was computed in the previous step. These PI controls deliver the reference values $\left(\vartheta_{r},\vartheta_{l}\right)$ for voltages to be applied at the motor armature terminals and represent the voltage profiles that must be tracked by the output voltage of the power stage.
3. Low-level control. In this level, two average controls based on differential flatness are designed. These controllers deliver the variables $\left(u_{av_{r}},u_{av_{l}}\right)$ that represent the signals to be applied to the transistors of the Buck power converters. Such controls ensure that voltage at the output of the power stage tracks the desired profile computed in the previous step.

With the aim of working as a whole, the controls designed in items (1), (2), and (3) are interconnected by using the hierarchical controller concept [7,8,9,10].

#### 2.2.1. High-Level Control

This subsection presents a kinematic control and the design of a computed-torque control for the mechanical structure subsystem of the DDWMR.

The configuration of the DDWMR can be described by the five generalized coordinates $q={\left[x,y,\varphi ,\theta_{r},\theta_{l}\right]}^{T}$, where (x,y) are the coordinates of $P_{0}$, φ is the heading angle of the robot, and $\theta_{r}$, $\theta_{l}$ are the angles of the right and left driving wheels (see Figure 1). Assuming that the wheels move without slippage, then the dynamic model of the DDWMR is given by [35]:

(1) $$\begin{array}{c} \dot{q}=S\left(q\right)\nu , \end{array}$$

(2) $$\begin{array}{c} M\left(q\right)\dot{\nu }+C\left(q,\dot{q}\right)\nu =B\left(q\right)\tau , \end{array}$$

where

$$\begin{array}{c} S\left(q\right)=\left(\begin{array}{cc} \frac{r}{2}\cos\varphi & \frac{r}{2}\cos\varphi \\ \frac{r}{2}\sin\varphi & \frac{r}{2}\sin\varphi \\ \frac{r}{2\ell } & -\frac{r}{2\ell }\\ 1 & 0\\ 0 & 1 \end{array}\right),\;M\left(q\right)=\left(\begin{array}{cc} \frac{r^{2}}{4\ell^{2}}\left(m\ell^{2}+I\right)+I_{w} & \frac{r^{2}}{4\ell^{2}}\left(m\ell^{2}-I\right)\\ \frac{r^{2}}{4\ell^{2}}\left(m\ell^{2}-I\right) & \frac{r^{2}}{4\ell^{2}}\left(m\ell^{2}+I\right)+I_{w} \end{array}\right), \end{array}$$

$$\begin{array}{c} C\left(q,\dot{q}\right)=\left(\begin{array}{cc} 0 & \frac{r^{2}}{2\ell }m_{c}d\dot{\varphi }\\ \frac{r^{2}}{2\ell }m_{c}d\dot{\varphi } & 0 \end{array}\right),\;B\left(q\right)=\left(\begin{array}{cc} 1 & 0\\ 0 & 1 \end{array}\right), \end{array}$$

and $\nu ={\left[\omega_{r},\omega_{l}\right]}^{T}$ represent the angular velocities of the driving wheels, previously defined. Whereas $\tau ={\left[\tau_{r},\tau_{l}\right]}^{T}$ is the input vector of torques $\tau_{r}$ and $\tau_{l}$ applied at the right and the left driving wheels, respectively. It was defined $m=m_{c}+2m_{w}$ and $I=m_{c}d^{2}+2m_{w}\ell^{2}+I_{c}+2I_{m}$. In the previous equations, and in the rest of the paper, the derivative with respect to time *t* will be specified by a “dot” or by d/dt.

The kinematic control is based on the kinematics of the DDWMR Equation (1). After defining $\upsilon =\sqrt{{\dot{x}}^{2}+{\dot{y}}^{2}}$ and $\omega =\dot{\varphi }$ the kinematics can be expressed as:

(3) $$\begin{array}{c} \begin{array}{cc} \dot{x} & =\upsilon \cos\varphi ,\\ \dot{y} & =\upsilon \sin\varphi ,\\ \dot{\varphi } & =\omega , \end{array} \end{array}$$

where υ and ω are the inputs.

The main objective of the kinematic control is to guarantee that $\left(x,y,\varphi \right)\to \left(x^{*},y^{*},\varphi^{*}\right)$. This is, the DDWMR must track a desired trajectory imposed by a reference DDWMR whose kinematic model is given by:

(4) $$\begin{array}{c} \begin{array}{cc} {\dot{x}}^{*} & =\upsilon^{*}\cos\varphi^{*},\\ {\dot{y}}^{*} & =\upsilon^{*}\sin\varphi^{*},\\ {\dot{\varphi }}^{*} & =\omega^{*}, \end{array} \end{array}$$

where the configuration of the reference DDWMR is given by the triplet $\left(x^{*},y^{*},\varphi^{*}\right)$ and the reference inputs are $\upsilon^{*}$ and $\omega^{*}$.

Based on Equations (3) and (4) the tracking error is defined as:

(5) $$\begin{array}{c} \left(\begin{array}{c} e_{1}\\ e_{2}\\ e_{3} \end{array}\right)=\left(\begin{array}{ccc} K_{2}\cos\varphi & K_{2}\sin\varphi & 0\\ -K_{2}\sin\varphi & K_{2}\cos\varphi & \alpha \\ 0 & 0 & 1 \end{array}\right)\left(\begin{array}{c} x^{*}-x\\ y^{*}-y\\ \varphi^{*}-\varphi \end{array}\right), \end{array}$$

where $K_{2}$ and α are positive constants. It is important to note that determinant of matrix in Equation (5) is $K_{2}^{2}$, meaning that the matrix is non-singular. After computing the time derivative of Equation (5), and considering Equations (3) and (4), the following error dynamics is obtained:

(6) $$\begin{array}{cc} \left(\begin{array}{c} {\dot{e}}_{1}\\ {\dot{e}}_{2}\\ {\dot{e}}_{3} \end{array}\right)\; & =\left(\begin{array}{cc} -K_{2} & e_{2}-\alpha e_{3}\\ 0 & -e_{1}-\alpha \\ 0 & -1 \end{array}\right)\left(\begin{array}{c} \upsilon \\ \omega \end{array}\right)+\left(\begin{array}{c} \upsilon^{*}K_{2}\cos e_{3}\\ \upsilon^{*}K_{2}\sin e_{3}+\alpha \omega^{*}\\ \omega^{*} \end{array}\right). \end{array}$$

According with [35], replacing ${\left[\upsilon ,\omega \right]}^{T}$ by the following control inputs is suitable for tracking purposes:

(7) $$\begin{array}{c} \begin{array}{cc} \upsilon_{f} & =\upsilon^{*}\cos e_{3}+K_{1}e_{1},\\ \omega_{f} & =\omega^{*}+\upsilon^{*}K_{2}e_{2}+K_{3}\sin e_{3}, \end{array} \end{array}$$

where $K_{1}$ and $K_{3}$ are positive constants. The control inputs Equation (7) allow that $\left(e_{1},e_{2},e_{3}\right)\to \left(0,0,0\right)$, as shown in [41], as long as $\alpha =1/K_{3}$ and $\upsilon^{*}>0$. Consequently, it is achieved that $\left(x,y,\varphi \right)\to \left(x^{*},y^{*},\varphi^{*}\right)$ when t→∞.

The computed-torque control is based on the dynamics of the DDWMR Equation (2). Such control is proposed as follows:

(8) $$\begin{array}{cc} \tau & =M\left(q\right)\left({\dot{\nu }}^{*}-K_{p}\tilde{\nu }-K_{i}\int_{0}^{t}\tilde{\nu }ds\right)+C\left(q,\dot{q}\right)\nu , \end{array}$$

where $\tau ={\left[\tau_{r},\tau_{l}\right]}^{T}$ and

(9) $$\begin{array}{c} \tilde{\nu }=\nu -\nu^{*}=\left(\begin{array}{c} \omega_{r}-\omega_{r}^{*}\\ \omega_{l}-\omega_{l}^{*} \end{array}\right), \end{array}$$

$$\begin{array}{c} K_{p}=\mathrm{diag}\left\{k_{p\upsilon },k_{p\omega }\right\},\;K_{i}=\mathrm{diag}\left\{k_{i\upsilon },k_{i\omega }\right\}, \end{array}$$

ν is defined by the following inverse transformation:

(10) $$\begin{array}{c} \left(\begin{array}{c} \omega_{r}\\ \omega_{l} \end{array}\right)=\left(\begin{array}{cc} \frac{1}{r} & \frac{\ell }{r}\\ \frac{1}{r} & -\frac{\ell }{r} \end{array}\right)\left(\begin{array}{c} \upsilon \\ \omega \end{array}\right), \end{array}$$

and $\nu^{*}$ is defined as:

(11) $$\begin{array}{c} \left(\begin{array}{c} \omega_{r}^{*}\\ \omega_{l}^{*} \end{array}\right)=\left(\begin{array}{cc} \frac{1}{r} & \frac{\ell }{r}\\ \frac{1}{r} & -\frac{\ell }{r} \end{array}\right)\left(\begin{array}{c} \upsilon_{f}\\ \omega_{f} \end{array}\right), \end{array}$$

where $\upsilon_{f}$ and $\omega_{f}$, given by Equation (7), are used.

After replacing the torque-control Equation (8) in the dynamics of the DDWMR Equation (2) the following error dynamics are obtained:

(12) $$\begin{array}{c} \dot{\tilde{\nu }}=-K_{p}\tilde{\nu }-K_{i}\int_{0}^{t}\tilde{\nu }ds. \end{array}$$

Note that Equation (12) can be expressed, according with Equations (10) and (11), as follows:

(13) $$\begin{array}{cc} \dot{\tilde{\upsilon }} & =-k_{p\upsilon }\tilde{\upsilon }-k_{i\upsilon }\int_{0}^{t}\tilde{\upsilon }ds, \end{array}$$

(14) $$\begin{array}{cc} \dot{\tilde{\omega }} & =-k_{p\omega }\tilde{\omega }-k_{i\omega }\int_{0}^{t}\tilde{\omega }ds, \end{array}$$

where

$$\tilde{\upsilon }=\upsilon -\upsilon_{f},\;\tilde{\omega }=\omega -\omega_{f},$$

and $k_{p\upsilon },k_{i\upsilon }$, $k_{p\omega }$, $k_{i\omega }$ are sufficiently large positive constants allowing that $\left(\tilde{\upsilon },\tilde{\omega }\right)\to \left(0,0\right)$.

With the aim of showing the stability in closed-loop of the kinematic control Equation (7) and the computed-torque control Equation (8) with the DDWMR, the following Lyapunov function candidate is proposed. This function is inspired by that proposed in [34], and it is complemented by some terms arising from rotative and translational velocity errors:

(15) $$\begin{array}{cc} V(e_{1},e_{2},e_{3},\tilde{\upsilon },\tilde{\omega }) & =\frac{1}{2}K_{1}e_{1}^{2}+\frac{1}{2}K_{1}e_{2}^{2}+\frac{K_{1}}{K_{2}}\left(1-\cos e_{3}\right)\\ \; & +\frac{1}{2}\left({\tilde{\upsilon }}^{2}+{\tilde{\omega }}^{2}\right)+\frac{1}{2}k_{i\upsilon }{\left(\int_{0}^{t}\tilde{\upsilon }ds\right)}^{2}+\frac{1}{2}k_{i\omega }{\left(\int_{0}^{t}\tilde{\omega }ds\right)}^{2}. \end{array}$$

The time derivative of *V* along the trajectories of the closed-loop system is given as:

$$\begin{array}{cc} \dot{V} & =-K_{1}^{2}e_{1}^{2}-K_{1}e_{1}\tilde{\upsilon }-\frac{K_{1}K_{3}}{K_{2}}\sin^{2}e_{3}-\frac{K_{1}}{K_{2}}\tilde{\omega }\sin e_{3}-k_{p\upsilon }{\tilde{\upsilon }}^{2}-K_{p\omega }{\tilde{\omega }}^{2},\\ \dot{V} & \leq -x^{T}Qx, \end{array}$$

where

$$\begin{array}{cc} Q & =\left(\begin{array}{cccc} K_{1}^{2} & 0 & -\frac{K_{1}}{2} & 0\\ 0 & \frac{K_{1}K_{3}}{K_{2}} & 0 & -\frac{K_{1}}{2K_{2}}\\ -\frac{K_{1}}{2} & 0 & K_{p\upsilon } & 0\\ 0 & -\frac{K_{1}}{2K_{2}} & 0 & K_{p\omega } \end{array}\right),\;x=\left[\right|e_{1}|,|\sin e_{3}\left|,\right|\tilde{\upsilon }\left|,\right|\tilde{\omega }{\left|\right]}^{T}. \end{array}$$

Matrix *Q* can be rendered positive definite if and only if its four principal minors are positive. This is always possible using large enough values for $K_{1}$, $K_{3}$, $K_{p\upsilon }$, $K_{p\omega }$. This ensures that $\dot{V}\leq 0$ and, hence, $e_{1},e_{2},e_{3},\tilde{\upsilon },\tilde{\omega }$, are bounded. Computing $\ddot{V}$ it is found that:

$$\begin{array}{cc} \ddot{V} & =-2K_{1}^{2}e_{1}{\dot{e}}_{1}-K_{1}{\dot{e}}_{1}\tilde{\upsilon }-K_{1}e_{1}\dot{\tilde{\upsilon }}-\frac{2K_{1}K_{3}}{K_{2}}{\dot{e}}_{3}\sin e_{3}\cos e_{3}-\frac{K_{1}}{K_{2}}\dot{\tilde{\omega }}\sin e_{3}\\ \; & \;-\frac{K_{1}}{K_{2}}\tilde{\omega }{\dot{e}}_{3}\cos e_{3}-2k_{p\upsilon }\tilde{\upsilon }\dot{\tilde{\upsilon }}-2K_{p\omega }\tilde{\omega }\dot{\tilde{\omega }}. \end{array}$$

Since $e_{1},e_{2},e_{3},\tilde{\upsilon },\tilde{\omega }$, are bounded so are ${\dot{e}}_{1},{\dot{e}}_{2},{\dot{e}}_{3}$ if $\upsilon^{*}$ and $\omega^{*}$ are bounded. Also note that $\dot{\tilde{\upsilon }}$ and $\dot{\tilde{\omega }}$ are bounded if $k_{p\upsilon },k_{i\upsilon },k_{p\omega },k_{i\omega }$ are positive. Hence, $\ddot{V}$ is bounded and Barbalat’s lemma ensures that $\dot{V}\to 0$ as t→∞, implying that $\left(e_{1},e_{3},\tilde{\upsilon },\tilde{\omega }\right)\to \left(0,0,0,0\right)$ locally. This result and considering that:

$$\begin{array}{c} \tilde{\omega }=\omega -\omega_{f}=\omega -\left(\omega^{*}+\upsilon^{*}K_{2}e_{2}+K_{3}\sin e_{3}\right)\to 0 \end{array}$$

also imply that $e_{2}\to 0$ if $\upsilon^{*}>0$. Thus, recalling that matrix in Equation (5) is nonsingular it is found that $x^{*}-x\to 0$, $y^{*}-y\to 0$, $\varphi^{*}-\varphi \to 0$.

It is stressed that conditions ensuring this result are the following: **(a)** τ is computed as in Equation (8), where Equations (7) and (11) are employed, **(b)** the controller gains $K_{1},K_{2},K_{3},K_{p\upsilon },K_{p\omega }$, must be chosen such that the four principal minors of matrix *Q* be positive, **(c)** the integral gains $K_{i\upsilon },K_{i\omega }$, may take any positive values, **(d)** $\upsilon^{*}$ and $\omega^{*}$ must be bounded functions of time with $\upsilon^{*}>0$ for all time.

#### 2.2.2. Medium-Level Control

This subsection presents a PI current control for the actuators subsystem of the DDWMR.

The dynamics of a DC motor, expressed in terms of its angular velocity ω, the armature current $i_{a}$, and voltage applied at the armature terminals ϑ is given by [41]:

(16) $$\begin{array}{c} \begin{array}{cc} L_{a}\frac{di_{a}}{dt} & =\vartheta -R_{a}i_{a}-k_{e}\omega ,\\ J\frac{d\omega }{dt} & =-b\omega +k_{m}i_{a}. \end{array} \end{array}$$

With the intention of achieving that the generated torque reaches torque τ defined in Equation (8), the torque equation

(17) $$\begin{array}{c} \tau =k_{m}i_{a}^{*} \end{array}$$

is employed to compute the required electric current $i_{a}^{*}$ to flow through the armature circuits. Then, the following PI electric current control is proposed

(18) $$\begin{array}{c} \vartheta_{k}=\gamma_{0}\left(i_{a_{k}}^{*}-i_{a_{k}}\right)+\gamma_{1}\int_{0}^{t}\left(i_{a_{k}}^{*}-i_{a_{k}}\right)d\tau , \end{array}$$

where subindex *k* stands for both *r* and *l*, whereas $\left(\gamma_{0},\gamma_{1}\right)$ are the positive control gains, to achieve $\left(i_{a_{r}},i_{a_{l}}\right)\to \left(i_{a_{r}}^{*},i_{a_{l}}^{*}\right)$. The theoretical justification behind this affirmation is the following.

Defining $e_{i}=i_{a}-i_{a}^{*}$ to represent the electric current error in any of the two motors, adding and subtracting some convenient terms, and using the Laplace transform with zero initial conditions, it is found that the feedback connection of Equations (16) and (18) results in

(19) $$\begin{array}{c} \begin{array}{cc} E_{i}\left(s\right) & =G_{1}\left(s\right)I_{a}^{*}\left(s\right)+G_{2}\left(s\right)\omega \left(s\right),\\ G_{1}\left(s\right) & =-\frac{R_{a}}{\gamma_{1}}s\frac{\left(s+R_{a}/L_{a}\right)}{R_{a}/L_{a}}\frac{\frac{\gamma_{1}}{L_{a}}}{s^{2}+\frac{R_{a}+\gamma_{0}}{L_{a}}s+\frac{\gamma_{1}}{L_{a}}},\\ G_{2}\left(s\right) & =-\frac{k_{e}}{\gamma_{1}}s\frac{\frac{\gamma_{1}}{L_{a}}}{s^{2}+\frac{R_{a}+\gamma_{0}}{L_{a}}s+\frac{\gamma_{1}}{L_{a}}}, \end{array} \end{array}$$

where $E_{i}\left(s\right)$, $I_{a}^{*}\left(s\right)$, ω(s), stand for the Laplace transforms of $e_{i}$, $i_{a}^{*}$, ω, respectively. Note that $G_{1}\left(s\right)$ and $G_{2}\left(s\right)$ are stable if $\gamma_{0}>0$ and $\gamma_{1}>0$.

**Figure 2:**
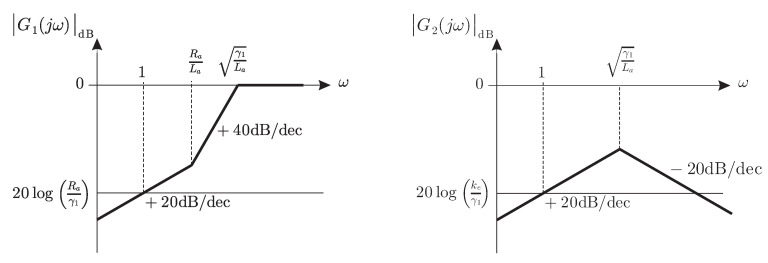
Frequency response to time varying $i_{a}^{*}$ and ω.

In Figure 2 the magnitude Bode diagram of $G_{1}\left(s\right)$ is presented. From this Bode diagram, it is concluded that the tracking error $e_{i}$ is small if the integral gain $\gamma_{1}$ is chosen to be large. Although this is valid for slow changing electric current profiles $i_{a}^{*}\left(t\right)$, i.e., for desired electric currents with low-frequency components, choosing larger integral gains allow to track electric current profiles with faster changes, i.e., desired electric currents with higher-frequency components. This is because the corner frequency $\sqrt{\gamma_{1}/L_{a}}$ is shifted to the right. Moreover, to avoid a large resonant peak, the proportional gain $\gamma_{0}$ must be chosen to be larger as the integral gain $\gamma_{1}$ is increased. Thus, it can be concluded that $i_{a}\left(t\right)$ remains close to $i_{a}^{*}\left(t\right)$ in steady-state, for an arbitrarily fast changing electric current profile $i_{a}^{*}\left(t\right)$, if both $\gamma_{0}$ and $\gamma_{1}$ are chosen to be large enough. Note that a 0[dB] magnitude for high frequencies in Figure 2 means that $|i_{a}-i_{a}^{*}\left|=\right|i_{a}^{*}|$, i.e., that $i_{a}\left(t\right)=0$.

On the other hand, Figure 2 also presents the magnitude Bode diagram of $G_{2}\left(s\right)$. Note that, because of the factor $k_{e}/\gamma_{1}$, attenuation for all frequencies is achieved as $\gamma_{1}$ is chosen to be large. However, $\gamma_{0}$ must also be large as $\gamma_{1}$ is large to avoid a large resonant peak. Hence, the effect of fast-changing external torque disturbances can be attenuated by using large values for both $\gamma_{0}$ and $\gamma_{1}$. Thus, the fact that $\left(i_{a_{r}},i_{a_{l}}\right)$ remain close to $\left(i_{a_{r}}^{*},i_{a_{l}}^{*}\right)$ in steady state, as stated above, is well justified.

#### 2.2.3. Low-Level Control

In this subsection, the design of a differential flatness-based control for the power stage subsystem of the DDWMR is presented.

The behavior of a DC/DC Buck power converter is governed by the following average model [42]:

(20) $$\begin{array}{c} \begin{array}{cc} L\frac{di}{dt} & =-v+Eu_{av},\\ C\frac{dv}{dt} & =i-\frac{v}{R}, \end{array} \end{array}$$

where $u_{av}\in \left[0,1\right]$ is the average input. When $u_{av}$ is replaced by the switched input u∈{0,1}, the mathematical model is known as switched model.

With the intention of accomplishing that $\left(v_{r},v_{l}\right)\to \left(\vartheta_{r},\vartheta_{l}\right)$, a differential flatness-based control is designed. For this purpose, the dynamics Equation (20) is expressed in terms of its flat output [43], S=v, as follows [44]:

(21) $$\begin{array}{c} u_{av}=\frac{LC}{E}\ddot{S}+\frac{L}{RE}\dot{S}+\frac{1}{E}S. \end{array}$$

A suitable proposal for $u_{av}$ is given by:

(22) $$u_{av}=\frac{LC}{E}\mu +\frac{L}{RE}\dot{S}+\frac{1}{E}S,$$

being μ an auxiliary control to be defined.

After introducing Equations (22) in (21) the following closed-loop dynamics is found:

(23) $$\begin{array}{c} {\ddot{S}}_{2}=\mu . \end{array}$$

In order to achieve that $S\to S^{*}$ as long as t→∞, being $S^{*}$ the desired voltage profile to be tracked by the output voltage of the Buck converter, the control signal μ is proposed as:

(24) $$\begin{array}{c} \mu ={\ddot{S}}^{*}-\beta_{2}\left(\dot{S}-{\dot{S}}^{*}\right)-\beta_{1}\left(S-S^{*}\right)-\beta_{0}\int_{0}^{t}\left(S-S^{*}\right)d\sigma . \end{array}$$

When Equation (24) is replaced in Equation (23) and the voltage tracking error is defined as $e_{c}=S-S^{*}$, the tracking error dynamics in closed-loop is obtained:

(25) $$\begin{array}{c} {\dddot{e}}_{c}+\beta_{2}{\ddot{e}}_{c}+\beta_{1}{\dot{e}}_{c}+\beta_{0}e_{c}=0, \end{array}$$

whose characteristic polynomial is given by:

(26) $$\begin{array}{c} p_{c}\left(s\right)=s^{3}+\beta_{2}s^{2}+\beta_{1}s+\beta_{0}. \end{array}$$

After equating Equation (26) with the following Hurwitz polynomial:

(27) $$\begin{array}{c} p_{d}\left(s\right)=\left(s+a\right)\left(s^{2}+2\xi \omega_{n}s+\omega_{n}^{2}\right), \end{array}$$

where $\left(a,\xi ,\omega_{n}\right)$ are positive constants; the control gains $\beta_{2}$, $\beta_{1}$, and $\beta_{0}$ are found to be:

$$\begin{array}{c} \beta_{2}=2\xi \omega_{n}+a,\;\beta_{1}=2\xi \omega_{n}a+\omega_{n}^{2},\;\beta_{0}=a\omega_{n}^{2}. \end{array}$$

#### 2.2.4. Dynamic Three-Level Tracking Controller

This section presents the design of a dynamic three-level tracking controller for the DDWMR. Such a controller is based on the hierarchical controller concept [7,8,9,10] and results from the interconnection of the controls previously designed in Section 2.2.1, Section 2.2.2 and Section 2.2.3.

After considering the kinematic and the dynamic mathematical models of the mechanical structure subsystem, the controls Equations (7) and (8) were proposed. These controls allow the DDWMR Equation (3) to track a desired trajectory imposed by the reference DDWMR Equation (4), i.e., $\left(\omega_{r},\omega_{l}\right)\to \left(\omega_{r}^{*},\omega_{l}^{*}\right)$ and $\left(x,y,\varphi \right)\to \left(x^{*},y^{*},\varphi^{*}\right)$. Since two DC motors are required to generate the desired torque in Equation (8), the current control Equation (18) was proposed for the actuators subsystem. Thus, the control inputs ensuring $\left(i_{a_{r}},i_{a_{l}}\right)\to \left(i_{a_{r}}^{*},i_{a_{l}}^{*}\right)$ are given by:

(28) $$\begin{array}{c} \vartheta_{r}=\gamma_{0_{r}}\left(i_{a_{r}}^{*}-i_{a_{r}}\right)+\gamma_{1_{r}}\int_{0}^{t}\left(i_{a_{r}}^{*}-i_{a_{r}}\right)d\tau , \end{array}$$

and

(29) $$\begin{array}{c} \vartheta_{l}=\gamma_{0_{l}}\left(i_{a_{l}}^{*}-i_{a_{l}}\right)+\gamma_{1_{l}}\int_{0}^{t}\left(i_{a_{l}}^{*}-i_{a_{l}}\right)d\tau , \end{array}$$

for the right and the left DC motors, respectively, with $i_{a_{r}}^{*}$ and $i_{a_{l}}^{*}$ defined as:

(30) $$\begin{array}{c} \begin{array}{c} i_{a_{r}}^{*}=k_{m}^{-1}\tau_{r}^{*},\\ i_{a_{l}}^{*}=k_{m}^{-1}\tau_{l}^{*}, \end{array} \end{array}$$

where Equation (17) has been used. In Equation (30) the following is considered:

(31) $$\begin{array}{c} \left(\tau_{r}^{*},\tau_{l}^{*}\right)=\left(\tau_{r},\tau_{l}\right), \end{array}$$

with $\left(\tau_{r},\tau_{l}\right)$ given in Equation (8). On the other hand, each DC motor is fed by a DC/DC Buck power converter and with the aim of achieving $\left(v_{r},v_{l}\right)\to \left(\vartheta_{r},\vartheta_{l}\right)$, the average control Equation (22) was designed. Hence, the right and the left Buck power converters are governed by the following average control inputs:

(32) $$\begin{array}{c} \begin{array}{cc} u_{av_{r}} & =\frac{L_{r}C_{r}}{E_{r}}\mu_{r}+\frac{L_{r}}{R_{r}E_{r}}{\dot{v}}_{r}+\frac{1}{E_{r}}v,\\ \mu_{r} & ={\ddot{v}}_{r}^{*}-\beta_{2_{r}}\left({\dot{v}}_{r}-{\dot{v}}_{r}^{*}\right)-\beta_{1_{r}}\left(v_{r}-{\dot{v}}_{r}^{*}\right)-\beta_{0_{r}}\int_{0}^{t}\left(v_{r}-v_{r}^{*}\right)d\tau , \end{array} \end{array}$$

and

(33) $$\begin{array}{c} \begin{array}{cc} u_{av_{l}} & =\frac{L_{l}C_{l}}{E_{l}}\mu_{l}+\frac{L_{l}}{R_{l}E_{l}}{\dot{v}}_{l}+\frac{1}{E_{l}}v_{l},\\ \mu_{l} & ={\ddot{v}}_{l}^{*}-\beta_{2_{l}}\left({\dot{v}}_{l}-{\dot{v}}_{l}^{*}\right)-\beta_{1_{l}}\left(v_{l}-{\dot{v}}_{l}^{*}\right)-\beta_{0_{l}}\int_{0}^{t}\left(v_{l}-v_{l}^{*}\right)d\tau , \end{array} \end{array}$$

respectively. In Equations (32) and (33) it is considered that:

(34) $$\begin{array}{c} \left(v_{r}^{*},v_{l}^{*}\right)=\left(\vartheta_{r},\vartheta_{l}\right). \end{array}$$

It is worth mentioning that the design of the average controls Equations (32) and (33) is based on the average mathematical model of the Buck power converter Equation (20). Consequently, the corresponding switched implementation $u_{r}$ and $u_{l}$ is carried out through the following Σ−Δ-modulators:

(35) $$\begin{array}{c} \begin{array}{cc} u_{r} & =\frac{1}{2}\left[1-\mathrm{sign}\left(e_{r}\right)\right],\\ \dot{e_{r}} & =u_{av_{r}}-u_{r}, \end{array} \end{array}$$

and

(36) $$\begin{array}{c} \begin{array}{cc} u_{l} & =\frac{1}{2}\left[1-\mathrm{sign}\left(e_{l}\right)\right],\\ \dot{e_{l}} & =u_{av_{l}}-u_{l}, \end{array} \end{array}$$

for the right and the left Buck power converters.

**Figure 3:**
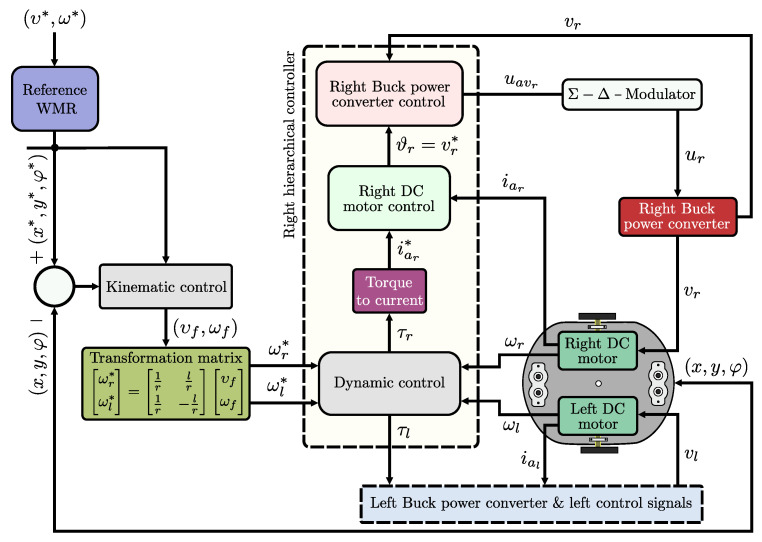
Block diagram of the dynamic three-level tracking controller.

In short, the switched controls Equations (35) and (36) achieve $\left(v_{r},v_{l}\right)\to \left(\vartheta_{r},\vartheta_{l}\right)$ which yields $\left(i_{a_{r}},i_{a_{l}}\right)\to \left(i_{a_{r}}^{*},i_{a_{l}}^{*}\right)$. Based on this, it is easily observed that the DDWMR will track a prescribed trajectory, i.e., $\left(\omega_{r},\omega_{l}\right)\to \left(\omega_{r}^{*},\omega_{l}^{*}\right)$ and $\left(x,y,\varphi \right)\to \left(x^{*},y^{*},\varphi^{*}\right)$. In Figure 3 the block diagram of the dynamic three-level tracking controller developed here is depicted.

## 3. Results from the Experimental Prototype in Closed-Loop

With the intention of highlighting the contribution of this paper, this section presents an experimental assessment of the dynamic three-level tracking controller designed in Section 2.2 with the hierarchical tracking controller reported in [9]. Likewise, the prototype of DDWMR used for the implementation of the controllers is described. Lastly, the experimental results are shown.

### 3.1. Controllers to Be Assessed

This subsection presents the generalities of the two controllers to be assessed. In this direction, the dynamic three-level tracking controller is firstly introduced, followed by the hierarchical tracking controller reported in [9].

#### 3.1.1. Dynamic Three-Level Tracking Controller

According with Section 2.2, the controller is composed of the following three levels:

- High-level. This control comprises the kinematic control given by,
  (37) $$\begin{array}{c} \begin{array}{cc} \upsilon_{f} & =\upsilon^{*}\cos e_{3}+K_{1}e_{1},\\ \omega_{f} & =\omega^{*}+\upsilon^{*}K_{2}e_{2}+K_{3}\sin e_{3}, \end{array} \end{array}$$
  and the computed-torque control proposed as,
  (38) $$\begin{array}{cc} \tau & =M\left(q\right)\left({\dot{\nu }}^{*}-K_{p}\tilde{\nu }-K_{i}\int_{0}^{t}\tilde{\nu }ds\right)+C\left(q,\dot{q}\right)\nu , \end{array}$$
  where $K_{1},K_{2},K_{3},K_{p}$, and $K_{i}$ are the controls gains.
- Medium-level. Corresponds to the PI current controls designed as,
  (39) $$\begin{array}{cc} \vartheta_{r} & =\gamma_{0_{r}}\left(i_{a_{r}}^{*}-i_{a_{r}}\right)+\gamma_{1_{r}}\int_{0}^{t}\left(i_{a_{r}}^{*}-i_{a_{r}}\right)d\tau , \end{array}$$
  (40) $$\begin{array}{cc} \vartheta_{l} & =\gamma_{0_{l}}\left(i_{a_{l}}^{*}-i_{a_{l}}\right)+\gamma_{1_{l}}\int_{0}^{t}\left(i_{a_{l}}^{*}-i_{a_{l}}\right)d\tau , \end{array}$$
  with $\left(\gamma_{0_{r}},\gamma_{1_{r}}\right)$ and $\left(\gamma_{0_{l}},\gamma_{1_{l}}\right)$ positive constants.
- Low-level. It was designed on the basis of the differential flatness concept as follows,
  (41) $$\begin{array}{c} \begin{array}{cc} u_{av_{r}} & =\frac{L_{r}C_{r}}{E_{r}}\mu_{r}+\frac{L_{r}}{R_{r}E_{r}}{\dot{v}}_{r}+\frac{1}{E_{r}}v_{r},\\ \mu_{r} & ={\ddot{v}}_{r}^{*}-\beta_{2_{r}}\left({\dot{v}}_{r}-{\dot{v}}_{r}^{*}\right)-\beta_{1_{r}}\left(v_{r}-{\dot{v}}_{r}^{*}\right)-\beta_{0_{r}}\int_{0}^{t}\left(v_{r}-v_{r}^{*}\right)d\tau , \end{array} \end{array}$$
  and
  (42) $$\begin{array}{c} \begin{array}{cc} u_{av_{l}} & =\frac{L_{l}C_{l}}{E_{l}}\mu_{l}+\frac{L_{l}}{R_{l}E_{l}}{\dot{v}}_{l}+\frac{1}{E_{l}}v_{l},\\ \mu_{l} & ={\ddot{v}}_{l}^{*}-\beta_{2_{l}}\left({\dot{v}}_{l}-{\dot{v}}_{l}^{*}\right)-\beta_{1_{l}}\left(v_{l}-{\dot{v}}_{l}^{*}\right)-\beta_{0_{l}}\int_{0}^{t}\left(v_{l}-v_{l}^{*}\right)d\tau , \end{array} \end{array}$$
  where the control gains $\left(\beta_{2_{r}},\beta_{1_{r}},\beta_{0_{r}}\right)$ and $\left(\beta_{2_{l}},\beta_{1_{l}},\beta_{0_{l}}\right)$ were found to be,
  $$\begin{array}{c} \beta_{2_{r}}=2\xi_{r}\omega_{n_{r}}+a_{r},\;\beta_{1_{r}}=2\xi_{r}\omega_{n_{r}}a_{r}+\omega_{n_{r}}^{2},\;\beta_{0_{r}}=a_{r}\omega_{n_{r}}^{2}, \end{array}$$
  and
  $$\begin{array}{c} \beta_{2_{l}}=2\xi_{l}\omega_{n_{l}}+a_{l},\;\beta_{1_{l}}=2\xi_{l}\omega_{n_{l}}a_{l}+\omega_{n_{l}}^{2},\;\beta_{0_{l}}=a_{l}\omega_{n_{l}}^{2}. \end{array}$$

#### 3.1.2. Hierarchical Tracking Controller

This controller was reported in [9] and also comprises three levels: high, medium, and low. In this regard, the high-level control is the same as control Equation (37), whereas the medium-level control was designed on the basis of the first order approximation of Equation (16) and was given by,

(43) $$\begin{array}{c} \vartheta_{r}=\frac{\delta_{r}+\zeta_{r}\omega_{r}}{\phi_{r}}, \end{array}$$

$$\begin{array}{c} \delta_{r}={\dot{\omega }}_{r}^{*}-k_{p_{r}}\left(\omega_{r}-\omega_{r}^{*}\right)-k_{i_{r}}\int_{0}^{t}\left(\omega_{r}-\omega_{r}^{*}\right)d\sigma_{r},\;\zeta_{r}=\frac{1}{\rho_{r}},\;\phi_{r}=\frac{K_{r}}{\rho_{r}}, \end{array}$$

and

(44) $$\begin{array}{c} \vartheta_{l}=\frac{\delta_{l}+\zeta_{l}\omega_{l}}{\phi_{l}}, \end{array}$$

$$\begin{array}{cc} \delta_{l} & ={\dot{\omega }}_{l}^{*}-k_{p_{l}}\left(\omega_{l}-\omega_{l}^{*}\right)-k_{i_{l}}\int_{0}^{t}\left(\omega_{l}-\omega_{l}^{*}\right)d\sigma_{l},\;\zeta_{l}=\frac{1}{\rho_{l}},\;\phi_{l}=\frac{K_{l}}{\rho_{l}}, \end{array}$$

while the gains $k_{p_{r}},k_{i_{r}},k_{p_{l}},k_{i_{l}}$ were defined as,

$$\begin{array}{c} k_{p_{r}}=2\xi_{1_{r}}\omega_{n_{1_{r}}},\;k_{i_{r}}=\omega_{n_{1_{r}}}^{2},\;k_{p_{l}}=2\xi_{1_{l}}\omega_{n_{1_{l}}},\;k_{i_{l}}=\omega_{n_{1_{l}}}^{2}. \end{array}$$

Related to the low-level control, it corresponds to the same used by the dynamic three-level tracking controller, i.e., Equations (41) and (42).

### 3.2. DDWMR Prototype and Connections Diagram

**Figure 4:**
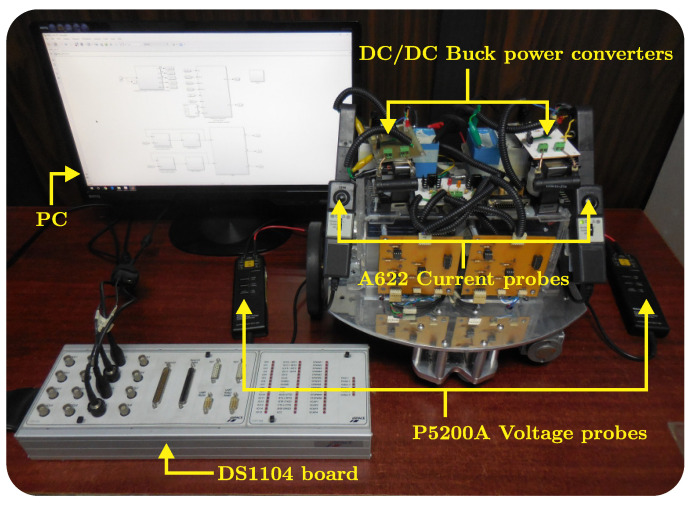
DDWMR prototype.

The controllers to be assessed are tested on a DDWMR prototype via Matlab-Simulink, the real-time interface ControlDesk, and a DS1104 board. The prototype is shown in Figure 4 and its dimensions are $390\times 10^{-3}\;m$ in length, $360\times 10^{-3}\;m$ in width, and $350\times 10^{-3}\;m$ in height, whereas its mass is 19kg. The wheels are steered by brushed DC motors GNM3150 + G2.6, provided with a 20:1 gearbox each, and are driven by DC/DC Buck power converters.

**Figure 5:**
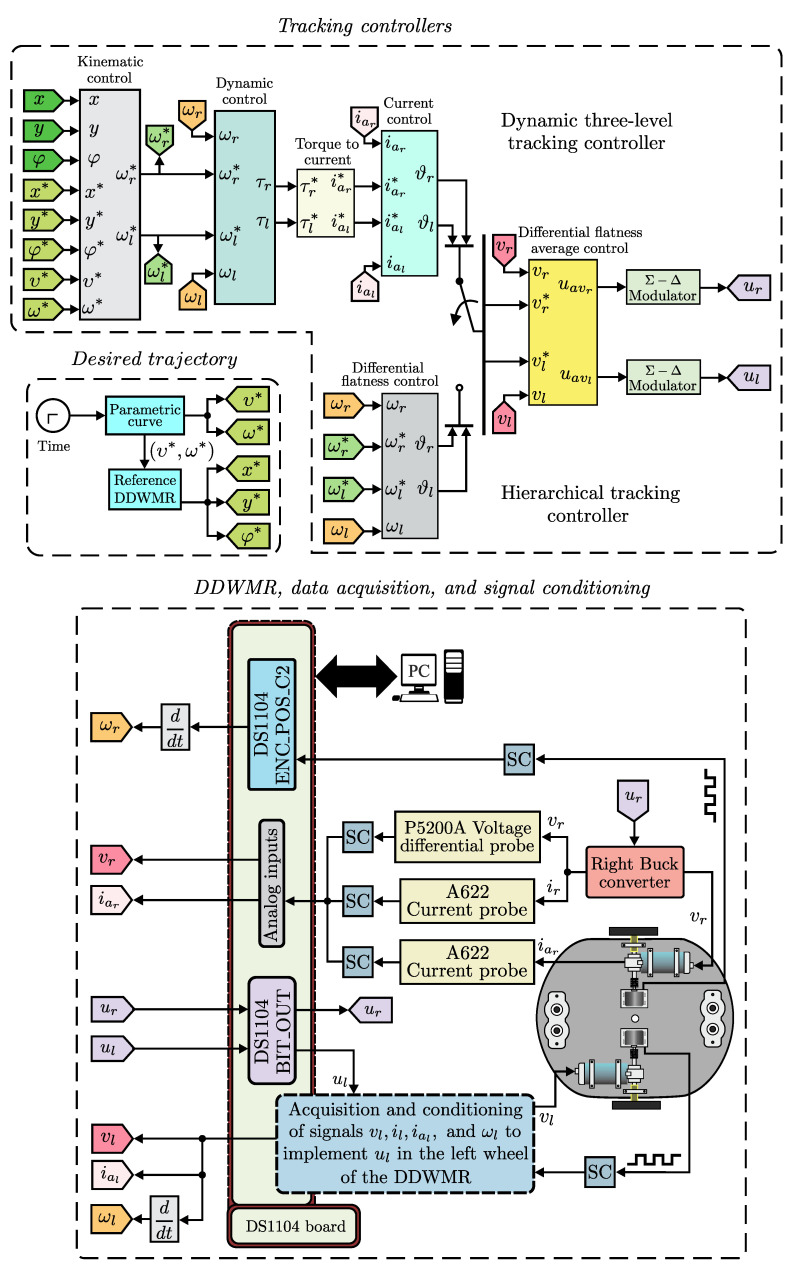
Connections diagram of the DDWMR in closed-loop with the tracking controllers.

On the other hand, Figure 5 depicts the connections diagram of the DDWMR in closed-loop. As can be observed, the DDWMR is tested with either the dynamic three-level tracking controller or the hierarchical tracking controller and Matlab-Simulink along with a DS1104 board.

In Figure 5 the following blocks were used:

(a) Tracking controllers. The dynamic three-level tracking controller Equations (37)–(42) and the hierarchical tracking controller Equation (37), Equations (41)–(44) reported in [9] were programmed in this block. Additionally, the following six sub-blocks were defined:
  (1) Kinematic control. Corresponds to the high level of both tracking controllers and is given by Equation (37). It requires the physical parameters of the mechanical structure defined as,
    r=0.075 m,2ℓ=0.40 m.
    The gains of this control for the dynamic three-level tracking controller are,
    $$K_{1}=0.1,\;K_{2}=5,\;K_{3}=1,$$
    whereas for the hierarchical tracking controller are,
    $$K_{1}=1.2,\;K_{2}=0.8,\;K_{3}=0.9.$$
  (2) Dynamic control. This control is also related to the high level of the dynamic three-level tracking controller and is given by Equation (38). In this control, the following parameters of the mechanical structure are required:
    $$m_{c}=15.8\;\mathrm{kg},\;m_{w}=2.47\;\mathrm{kg},\;I_{c}=0.135\;\mathrm{kg}\cdot m^{2},\;I_{w}=0.00375\;\mathrm{kg}\cdot m^{2},\;I_{m}=0.0015\;\mathrm{kg}\cdot m^{2},$$
    while the gains were selected as,
    $$k_{p\upsilon }=50,\;k_{i\upsilon }=140,\;k_{p\omega }=30,\;k_{i\omega }=150.$$
  (3) Torque to current. In this sub-block the mathematical transformations from torque to current Equation (30) are programmed, where $k_{m_{r}}=k_{m_{l}}=1.748\;N\cdot \;m/A.$
  (4) Current control. Corresponds to the medium level of the dynamic three-level tracking controller and is given by Equations (39) and (40), while its gains were proposed as follows:
    $$\gamma_{0_{r}}=2.8,\;\gamma_{1_{r}}=15,\;\gamma_{0_{l}}=2,\;\gamma_{1_{l}}=10.$$
  (5) Differential flatness control. This control is associated with the medium level of the hierarchical tracking controller and is defined by Equations (43) and (44). In these equations the following parameters are required:
    $$\rho_{r}=20\times 10^{-3},\;K_{r}=634\times 10^{-3},\;\rho_{l}=20\times 10^{-3},\;K_{l}=580\times 10^{-3}.$$
    The gains of this control were found by proposing the following parameters:
    $$\xi_{1_{r}}=1.63,\;\omega_{n_{1_{r}}}=24.5,\;\xi_{1_{l}}=1.25,\;\omega_{n_{1_{l}}}=20.$$
  (6) Differential flatness average control. This one corresponds to the low level of both tracking controllers and is given by Equations (41) and (42). This block contains the following nominal parameters of the power converters:
    $$R_{r}=R_{l}=100\;\Omega ,\;C_{r}=C_{l}=220\;\mu F,\;L_{r}=10.129\;mH,\;L_{l}=10.6\;mH,\;E_{r}=E_{l}=28\;V.$$
    Also, the parameters of the corresponding gains are programmed in here. For the dynamic three-level tracking controller the parameters were proposed as,
    $$a_{r}=300,\;\xi_{r}=850,\;\omega_{n_{r}}=800,\;a_{l}=350,\;\xi_{l}=800,\;\omega_{n_{l}}=600,$$
    while for the hierarchical tracking controller, the following parameters were defined:
    $$a_{r}=120,\;\xi_{r}=150,\;\omega_{n_{r}}=80,\;a_{l}=180,\;\xi_{l}=150,\;\omega_{n_{l}}=250.$$
    The synthesis of this control was carried out through the Σ−Δ-modulators Equations (35) and (36).
(b) Desired trajectory. The trajectory to be tracked by the DDWMR, when either the dynamic three-level tracking controller or the hierarchical tracking controller is used, is proposed as a circle path given by,
  (45) $$\begin{array}{c} \begin{array}{cc} x^{*} & =A\sin\omega t,\\ y^{*} & =A(1-\cos\omega t),\\ \varphi^{*} & =\omega t,\\ \upsilon^{*} & =A\omega ,\\ \omega^{*} & =\omega , \end{array} \end{array}$$
  where A=1.5m, ω=2πfrad/s, and f=1/THz, being $T=K_{s}\;s$ and $K_{s}$ a constant value to be defined.
(c) DDWMR, data acquisition, and signal conditioning. Here, the connections between the DDWMR prototype and the DS1104 board are presented. The data acquisition of voltages $\left(v_{r},v_{l}\right)$, currents $\left(i_{r},i_{l},i_{a_{r}},i_{a_{l}}\right)$, and angular velocities $\left(\omega_{r},\omega_{l}\right)$ is carried out through Tektronix P5200A voltage probes, Tektronix A622 current probes, and Autonics E50S8-1000 incremental encoders, respectively. In addition, a signal conditioning (SC) is executed in each signal.

### 3.3. Experimental Results

This subsection presents a visual assessment, through experimental results, of the dynamic three-level tracking controller proposed in this paper, Equations (37)–(42), and the hierarchical tracking controller, Equations (37), (41)–(44), reported in [9]. Note that, in such experiments, the results associated with the dynamic three-level tracking controller are labeled as $y_{d}\left(x_{d}\right)$, $\varphi_{d}$, $\omega_{r_{d}}$, $\omega_{l_{d}}$, $v_{r_{d}}$, $v_{l_{d}}$, $i_{a_{r}}$, $i_{a_{l}}$, $i_{r_{d}}$, and $i_{l_{d}}$, while the results related to the hierarchial tracking controller correspond to $y_{k}\left(x_{k}\right)$, $\varphi_{k}$, $\omega_{r_{k}}$, $\omega_{l_{k}}$, $v_{r_{k}}$, $v_{l_{k}}$, $i_{r_{k}}$, and $i_{l_{k}}$. On the other hand, the results of the tracking errors associated with the dynamic three-level tracking controller correspond to $e_{x_{d}}$, $e_{y_{d}}$, and $e_{\varphi_{d}}$. These errors have been defined as,

$$\begin{array}{c} e_{x_{d}}=x^{*}-x_{d},\;e_{y_{d}}=y^{*}-y_{d},\;e_{\varphi_{d}}=\varphi^{*}-\varphi_{d}. \end{array}$$

While the results of the tracking errors related to the hierarchical tracking controller are $e_{x_{k}}$, $e_{y_{k}}$, and $e_{\varphi_{k}}$, and have been defined as,

$$\begin{array}{c} e_{x_{k}}=x^{*}-x_{k},\;e_{y_{k}}=y^{*}-y_{k},\;e_{\varphi_{k}}=\varphi^{*}-\varphi_{k}. \end{array}$$

The rest of variables are the desired trajectories to be tracked and were previously defined.

With the aim of enhancing even more the contribution of this paper, the experimental results in closed-loop contemplated three cases: (i) when nominal values of all parameters associated with the DDWMR were considered, (ii) when abrupt variations appeared in loads $\left(R_{l},R_{r}\right)$ of the Buck power converters, and (iii) when abrupt variations were introduced in power supplies $\left(E_{l},E_{r}\right)$ of the Buck power converters.

#### 3.3.1. Experiment 1: Considering Nominal Values

**Figure 6:**
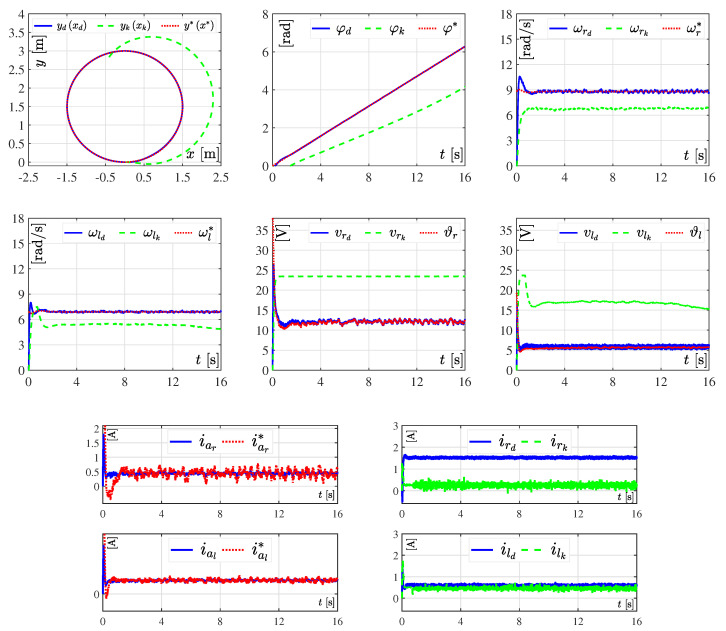
Experimental results in closed-loop when considering nominal values in all parameters of the DDWMR.

**Figure 7:**
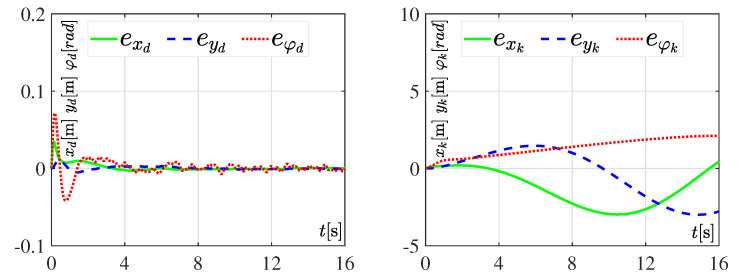
Tracking errors when considering nominal values in all parameters of the DDWMR.

The performance of the DDWMR in closed-loop, with either the dynamic three-level tracking controller or the hierarchical tracking controller, when no kind of variations appeared in its parameters is presented in Figure 6, whereas Figure 7 shows the tracking errors related to *x*, *y*, and φ, for both controllers. For these experiments, the value of parameter $K_{s}=16\;s$, meaning the tracking task was solved in 16 s. The reason for choosing such a value is because when $K_{s}<16\;s$ neither the dynamic three-level controller nor the hierarchical controller solved the control objective. However, when $K_{s}=16\;s$ the dynamic one achieves $\left(x,y,\varphi \right)\to \left(x^{*},y^{*},\varphi^{*}\right)$ but not the hierarchical one, since this latter did not consider the dynamic model of the subsystem mechanical structure, as can be observed in Figure 6 and Figure 7. This, in turn, means that the DDWMR in closed-loop with the hierarchial tracking controller could not drive high speeds. Thus, the good performance of the proposed approach was validated, since all signals tracked their corresponding desired trajectories, i.e., the control objective was achieved.

#### 3.3.2. Experiment 2: Variations in Loads $R_{r}$ and $R_{l}$

**Figure 8:**
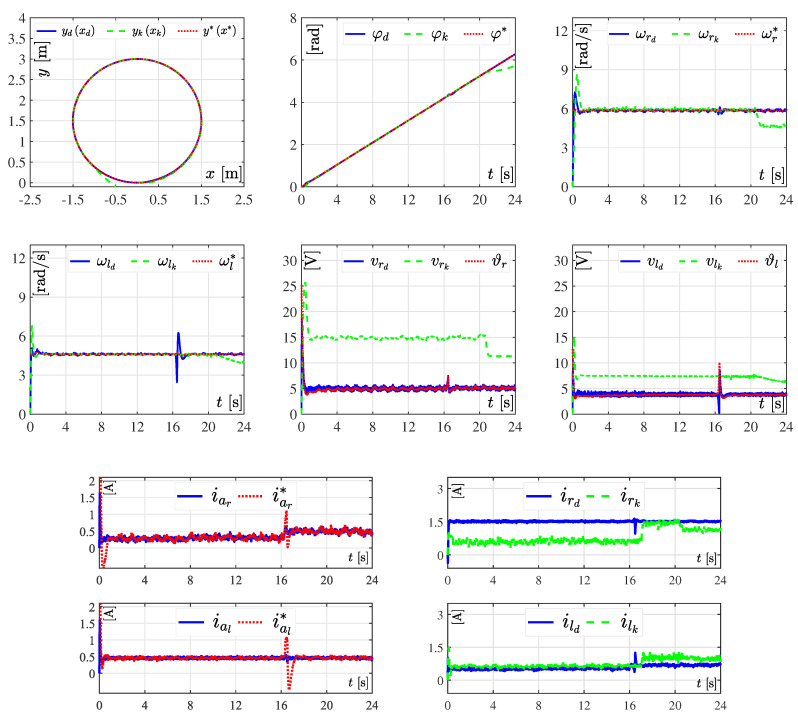
Experimental results in closed-loop with abrupt changes in $R_{r}$ and $R_{l}$. The results of the dynamic three-level tracking controller are labeled as $y_{d}\left(x_{d}\right)$, $\varphi_{d}$, $\omega_{r_{d}}$, $\omega_{l_{d}}$, $v_{r_{d}}$, $v_{l_{d}}$, $i_{a_{r}}$, $i_{a_{l}}$, $i_{r_{d}}$, and $i_{l_{d}}$, while the results associated with the hierarchical tracking controller correspond to $y_{k}\left(x_{k}\right)$, $\varphi_{k}$, $\omega_{r_{k}}$, $\omega_{l_{k}}$, $v_{r_{k}}$, $v_{l_{k}}$, $i_{r_{k}}$, and $i_{l_{k}}$.

With the aim of assessing the robustness of both tracking controllers, the variations of Table 2 for loads $R_{r}$ and $R_{l}$ were introduced. In these results the value of parameter $K_{s}=24\;s$, i.e., the motion of the DDWMR was slightly slower compared to the previous experiment, as depicted in Figure 8.

**Figure 9:**
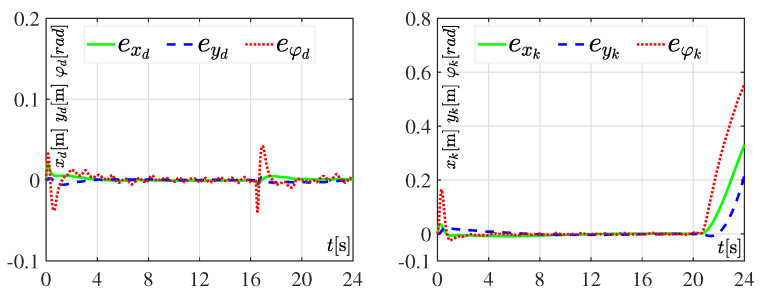
Tracking errors when the abrupt changes in $R_{r}$ and $R_{l}$ (Table 2) are considered. In these results, the tracking errors associated with the dynamic three-level tracking controller are denoted by $e_{x_{d}}$, $e_{y_{d}}$, and $e_{\varphi_{d}}$, while for the hierarchical tracking controller the corresponding errors are represented by $e_{x_{k}}$, $e_{y_{k}}$, and $e_{\varphi_{k}}$.

As it can be observed in Figure 8 and Figure 9, tracking errors appeared when using the hierarchical tracking controller, despite its robustness and the slower movement of the DDWMR. This is due to the controller does not consider the dynamics of the mechanical structure. In contrast, the dynamic three-level tracking controller did achieve $\left(x,y,\varphi \right)\to \left(x^{*},y^{*},\varphi^{*}\right)$, validating its good performance and robustness. It is worth emphasizing that although both tracking controllers were indeed robust, the hierarchical one was limited when selecting the trajectory to be tracked by the DDWMR. This means that when using the kinematics of the mechanical structure in control design, the DDWMR could fail if curve trajectories were imposed at high speeds.

#### 3.3.3. Experiment 3: Abrupt Changes in Power Supplies $E_{r}$ and $E_{l}$

**Figure 10:**
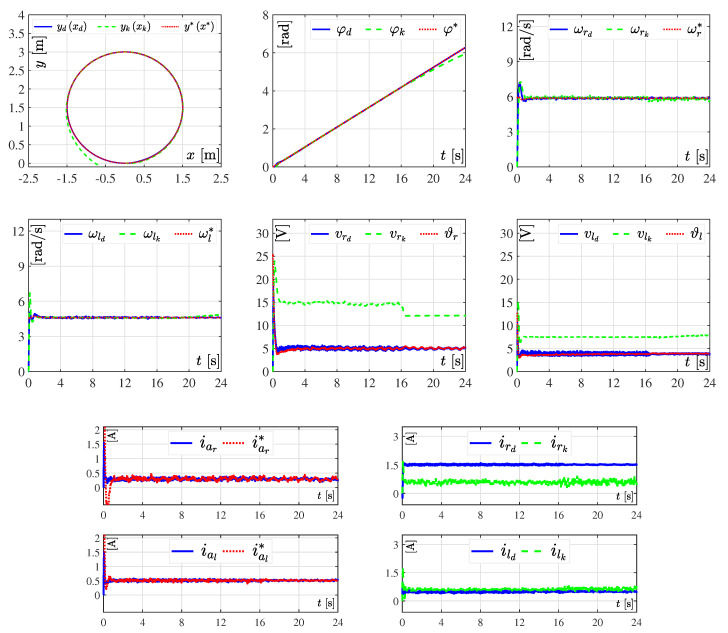
Experimental results in closed-loop when abrupt changes in $E_{r}$ and $E_{l}$ are introduced. The results associated with the dynamic three-level tracking controller are labeled as $y_{d}\left(x_{d}\right)$, $\varphi_{d}$, $\omega_{r_{d}}$, $\omega_{l_{d}}$, $v_{r_{d}}$, $v_{l_{d}}$, $i_{a_{r}}$, $i_{a_{l}}$, $i_{r_{d}}$, and $i_{l_{d}}$, while the results related to the hierarchical tracking controller correspond to $y_{k}\left(x_{k}\right)$, $\varphi_{k}$, $\omega_{r_{k}}$, $\omega_{l_{k}}$, $v_{r_{k}}$, $v_{l_{k}}$, $i_{r_{k}}$, and $i_{l_{k}}$.

The performance and robustness of both tracking controllers are assessed when considering the abrupt changes of Table 3 for power supplies $E_{r}$ and $E_{l}$. Similar to the previous experiment, here the value of parameter $K_{s}=24\;s$. The results of the experiment are shown in Figure 10.

**Figure 11:**
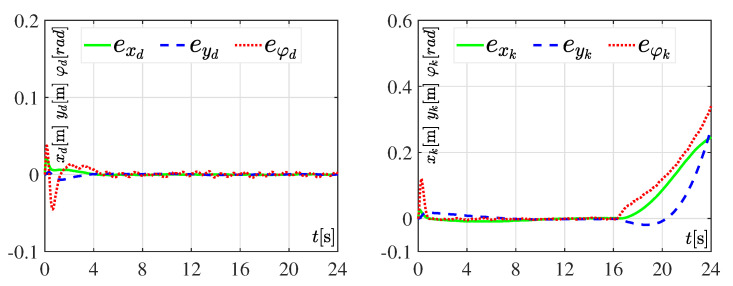
Tracking errors of the system in closed-loop, when the abrupt changes in $E_{r}$ and $E_{l}$ (Table 3) are considered. In these graphics the tracking errors associated with the dynamic three-level tracking controller are denoted as $e_{x_{d}}$, $e_{y_{d}}$, and $e_{\varphi_{d}}$, while the errors related to the hierarchial tracking controller are represented by $e_{x_{k}}$, $e_{y_{k}}$, and $e_{\varphi_{k}}$.

According with Figure 10 the DDWMR in closed-loop with the hierarchical tracking controller failed, once more, to track the desired trajectory, i.e., tracking errors appear (see Figure 11), while the dynamic three-level tracking controller achieved, again, the control goal $\left(x,y,\varphi \right)\to \left(x^{*},y^{*},\varphi^{*}\right)$, validating both its good performance and robustness.

In brief, the experimental results presented in Figure 6, Figure 7, Figure 8, Figure 9, Figure 10 and Figure 11 demonstrate that the dynamic three-level tracking controller successfully solved the trajectory tracking task. In this regard, two aspects must be highlighted: (1) no matter whether or not variations were considered in some parameters of the DDWMR, $\left(x,y,\varphi \right)\to \left(x^{*},y^{*},\varphi^{*}\right)$ was achieved; and (2) the DDWMR in closed-loop with such a controller was capable of solving the task in a smaller time, compared with the hierarchical tracking controller.

## 4. Conclusions and Future Work

The design of a novel dynamic three-level tracking controller for a DDWMR has been presented in this paper. The controller considers in its design the dynamic model of the mechanical structure, actuators, and power stage. Hence, three levels were proposed for the controller: (1) the high level, for the mechanical structure, is composed by a dynamic control and a kinematic one; (2) the medium level, for the actuators, is a PI current control; and (3) the low level, for the power stage, is a differential flatness-based control.

With the aim of showing the feasibility and robustness of the proposed controller, an assessment with the hierarchical tracking controller reported in [9] was carried out through experiments on a DDWMR via Matlab-Simulink, the real-time interface ControlDesk, and a DS1104 board. Based on the experimental results, the dynamic three-level tracking controller solves the tracking task, i.e., $\left(e_{x_{d}},e_{y_{d}},e_{\varphi_{d}}\right)\to \left(0,0,0\right)$, even when abrupt variations are introduced in some parameters of the DDWMR (see Figure 7, Figure 9 and Figure 11), while the hierarchical tracking controller failed to solve the control objective, since it was designed on the basis of the kinematic model of the mechanical structure. This latter means that high speeds cannot be considered when such a model is used.

Finally, future research will be focused on solving the obstacle avoidance and the path following control tasks by using the approach presented in this paper. On the other hand, and with the aim of possibly generating a new direction for this research topic, it would be interesting to implement some kind of learning technology such as machine learning [45] or Internet of things (IoT) [46,47]. Also, using wireless network sensors (WSNs) [48] could be another interesting path for highlighting even more the research proposed in this paper.

## Figures and Tables

**Table 1:** Tracking controls reported in literature for differential drive wheeled mobile robots (DDWMRs).

	Mathematical Model of the	
	*Mechanical Structure*	
**Subsystem**	**Kinematics**	**Dynamics**
MS	[15,16,17,18,19,20,21,22,23,24,25,26,27,28,29]	[36,37,38]
MS + A	[33]	[39,40]
MS + A + PS	[7,8,9,10]	—

**Table 2:** Abrupt changes in $R_{r}$ and $R_{l}$.

$R_{r}$		$R_{l}$	
$R_{r}$	t<8s	$R_{l}$	t<8s
$65\%\;R_{r}$	8s≤t<16s	$65\%\;R_{l}$	8s≤t<16s
$20\%\;R_{r}$	16s≤t	$20\%\;R_{l}$	16s≤t

**Table 3:** Abrupt changes in $E_{r}$ and $E_{l}$.

$E_{m_{r}}$		$E_{m_{l}}$	
$E_{r}$	t<8s	$E_{l}$	t<8s
$85\%\;E_{r}$	8s≤t<16s	$85\%\;E_{l}$	8s≤t<16s
$50\%\;E_{r}$	16s≤t	$50\%\;E_{l}$	16s≤t
